# The Medical Congress

**Published:** 1887-03

**Authors:** 


### Editorial.

### THE MEDICAL CONGRESS.

In "Current News" will be found the list of the officers and committees of the Section of Dental and Oral Surgery of the coming Congress. A considerable number of them have been appointed but recently. For those who have responsible places upon committees there is but little time left to do a great deal of work, and if a creditable exhibition is made, it will be because the members have shown extraordinary diligence. Since it is agreed that all shall unite in making everything possible of the section, not a day should be lost.

There still seems to be some misapprehension in the minds of dentists concerning the method of admission to the Congress. Every one should know exactly the status he will occupy. It has been asserted that the door has been opened yet wider, and that now dentists can enter upon conditions that were impossible but a short time since, or if this has not yet been done that it at once should be. A little reflection will convince any one that this is impracticable. This is a *Medical* Congress, and none but medical men can become members, unless as specially invited guests, or members by invitation. No degree save that of the M. D. *can* be recognized. A man may be an LL. D., D. D., Ph. D., S. T. D., B. S., B. A., M. A., may possess any or all these degrees, but they will not admit him to membership in the Congress. He may have all the learning of the schools, he may know more of medicine than Hippocrates himself, but if he have not the indispensable M. D., or its foreign equivalent, he is not actually a member of the medical profession, which makes that degree a *sine qua non*, and he cannot, except by special invitation, become a member of a Medical Congress. This has always been the rule in former sessions, and it cannot be changed by the representatives of one nationality, even though the meeting for the year is with them.

When the Congress met in London, the Prince of Wales, the Crown Prince of Prussia, the Archbishop of Canterbury and the other high church and state dignitaries became members, not through any degrees or titles which they held, but by special invitation. The President of the United States cannot become a member of the Congress of 1887 in any other way. In a Medical Congress no degree is equal to the medical degree, and no diploma, save that of a regular medical school, can by any possibility be recognized, however much it might be desired, because that degree is the only qualification required.

It has been claimed that societies should be permitted to name those who are to become members of the Congress. As well might it be claimed that medical societies might elect representatives to a Presbyterian synod, or a Methodist conference. Those bodies are for members of the clergy alone. This is a Congress of another profession—the medical—and none but those who belong to that profession are entitled to membership. It should be comprehended that the Congress is not a delegated body. Medical men are not elected or nominated. They join simply as members of the medical profession, and no one, no matter what may be the sum of his knowledge, can have a prescriptive right to membership unless he belongs to that profession. A synod, a conference, or a medical congress may invite those who are not of their profession to a place, and in this way laymen might become members, but in no other way, unless special provision for lay membership exists in their constitution. Were it otherwise, the very boundaries which limit the professions would be obliterated, and there would no longer exist any professions. It seems to us inconceivable that any one should for a moment imagine or dream of the possibility of the acknowledgment of any degree by a medical congress, save the exclusively medical one, or conceive the absurdity of the admission of laymen into a society whose only qualification is a medical degree, unless it be as invited guests. What would be thought of the physician who should present himself at a meeting of the American Dental Association, and on the strength of his medical practice—he being, for all any one knew, the veriest quack in existence—should demand admission to membership? Would not his assurance be likely to receive a severe check? One has but to reverse the picture to see what must of necessity be the case with the Medical Congress.

Nor should it be conceived that the invited members occupy an inferior or subordinate position. The list will comprise all the dignitaries of the land, who are members. The President of the United States will have precisely that status. The Secretary of State will be an invited member. It is an extraordinary concession that dentists who are without the medical degree will, very many of them, be invited, and will have every privilege in the Section of Dental and Oral Surgery that the oldest member has. Some of those who are officers of the section are without the M. D., but they obtain their membership precisely as will any others without that qualification. As members by special invitation, their position is much more honorable than though they joined by prescriptive right.

Not every one who is a dentist will be invited. This is a matter that is quite beyond the control of the Section. The Congress as a whole decides how many, aside from those who are entitled to membership through belonging to the medical profession, shall be invited. It allots a definite number to each State. This it has the right to do, and it assumes that right and acts upon it. We might desire that the rules of the Congress were otherwise, but we must accept them as they are, and no power, save that of the Congress in full session, can abrogate or modify those rules. They are a part of the organic laws of the organization, and must be obeyed.

Every dentist, then, who is without the medical degree, can only become a member of the Congress by receiving a special invitation. It has always been so, and probably always will remain so. We plainly stated that in an editorial in this journal more than two years ago, and as a member of the Congress which met in London we felt that we had some knowledge of the subject of which we spoke. In the number for February, 1885, we said : "As a medical body it is, of course, quite impossible for the Congress to recognize any but their own distinguishing medical degree of M. D. A reference to the first rule will convince any one of this," and in the succeeding number, we said that some provision must and would be made for the admission of those who are without this degree. The only provision possible, and the very one which has been active in former sessions, has been made, and therewith we must rest content. If ever a dental congress is called to meet in America, it will be our province to determine the conditions of membership, and then those who are only medical men must, if they would become a part of such a congress, come in by the door which we shall open. Certainly, they can have no prescriptive rights, unless the call is for dentists *and* general practitioners, any more than they would have in a congress of ophthalmologists or dermatologists.

We have thought it but just that this subject should be fully comprehended, for there are those who insist that every practicing dentist should be admitted without question, whereas such a thing is an utter impossibility, no one having the power to throw down the barrier. Dentists must come in as does every one else—either as medical men or by special invitation, the privileges of both in the Section being identical.

